# Surviving Elderly Patients with Head-and-Neck Squamous Cell Carcinoma—What Is the Long-Term Quality of Life after Curative Radiotherapy?

**DOI:** 10.3390/cancers13061275

**Published:** 2021-03-13

**Authors:** Alexander Rühle, Erik Haehl, Tobias Kalckreuth, Raluca Stoian, Simon K. B. Spohn, Tanja Sprave, Constantinos Zamboglou, Eleni Gkika, Andreas Knopf, Anca-Ligia Grosu, Nils H. Nicolay

**Affiliations:** 1 Department of Radiation Oncology, University of Freiburg—University Medical Center Freiburg, Robert-Koch-Str. 3, 79106 Freiburg, Germany; alexander.ruehle@uniklinik-freiburg.de (A.R.); erik.haehl@uniklinik-freiburg.de (E.H.); tobias.kalckreuth@uniklinik-freiburg.de (T.K.); raluca.stoian@uniklinik-freiburg.de (R.S.); simon.spohn@uniklinik-freiburg.de (S.K.B.S.); tanja.sprave@uniklinik-freiburg.de (T.S.); constantinos.zamboglou@uniklinik-freiburg.de (C.Z.); eleni.gkika@uniklinik-freiburg.de (E.G.); anca.grosu@uniklinik-freiburg.de (A.-L.G.); 2 German Cancer Consortium (DKTK) Partner Site Freiburg, German Cancer Research Center (dkfz), Neuenheimer Feld 280, 69120 Heidelberg, Germany; 3 Department of Otorhinolaryngology, University of Freiburg—University Medical Center Freiburg, Killianstr. 5, 79106 Freiburg, Germany; andreas.knopf@uniklinik-freiburg.de

**Keywords:** head-and-neck cancer, head-and-neck squamous cell carcinoma, radiotherapy, chemotherapy, quality of life, patient-reported outcomes, elderly patients, geriatric patients

## Abstract

**Simple Summary:**

As the long-term effects of radiotherapy on the quality of life (QoL) of elderly (≥65 years) patients with head-and-neck squamous cell carcinoma (HNSCC) are not well understood, we examined the QoL of surviving elderly HNSCC patients who were treated with radiotherapy. In this cross-sectional study, long-term quality of life (QoL) at ≥1 year after radiotherapy completion was comparable to the general German population of the same age and gender. Patients whose HNSCC was induced by human papillomavirus (HPV) exhibited a superior long-term global QoL than elderly patients with HPV-negative tumors. Interestingly, concomitant chemotherapy administration did not deteriorate patients’ global QoL in the long-term. We did not observe differences in patients’ health-related QoL in dependence of the treatment (definitive versus adjuvant (chemo)radiotherapy). Our data are important for clinicians treating elderly HNSCC patients as well as for elderly HNSCC patients themselves.

**Abstract:**

The effects of radiotherapy on the long-term quality of life (QoL) of surviving elderly HNSCC patients are not well understood, therefore, we analyzed QoL in this population. A cross-sectional analysis was performed at a tertiary cancer center to assess long-term QoL in elderly HNSCC patients. Eligible patients were ≥65 years at the time of treatment who had to be alive for ≥1 year after radiotherapy and without current anti-cancer treatment. QoL and patient satisfaction were assessed using the EORTC QLQ-C30, QLQ-H&N35 and ZUF-8 questionnaires, respectively, and treatment-related toxicities were graded according to CTCAE (Common Terminology Criteria of Adverse Effects) v.5.0. Seventy-four patients met the inclusion criteria, of which 50 consented to participate. Median time between radiotherapy and QoL assessment was 32 months (range 12–113). The QLQ-C30 global QoL median amounted to 66.7 points (interquartile range (IQR) 50.0–83.3), which was comparable to the age- and gender-adjusted German population (median 65.3). Median global QoL was similar between patients undergoing definitive (75.0, IQR 50.0–83.3) and adjuvant (chemo)radiotherapy (66.7, IQR 41.7–83.3, *p* = 0.219). HPV-positive HNSCC patients had superior global QoL after radiotherapy than their HPV-negative counterparts (*p* < 0.05), and concomitant chemotherapy did not influence the long-term QoL (*p* = 0.966). Median global QoL did not correspond with physician-assessed highest-graded chronic toxicities (*p* = 0.640). The ZUF-8 ranged at 29 points in median (IQR 27–31), showing high patient satisfaction. Surviving elderly HNSCC patients treated by radiotherapy exhibit a relatively high long-term global QoL which is a relevant information for clinicians treating elderly HNSCC patients.

## 1. Introduction

Head-and-neck squamous cell carcinoma (HNSCC) constitutes a frequent malignancy and causes more than 300,000 deaths per year globally [1]. The average age of HNSCC patients at the time of diagnosis ranges between 60 and 70 years, and the percentage of elderly HNSCC patients is believed to significantly increase over the next decades [2,3,4,5]. Both clinical and tumor-associated characteristics differ between younger and elderly HNSCC patients; for instance, the female-male-distribution is shifted towards female patients and the prevalence of HPV-related carcinomas is lower in elderly HNSCC patients [5]. In addition, the benefit of both concomitant chemotherapy and epidermal growth factor receptor antibody treatment is continuously decreasing with higher age [6,7]. As elderly HNSCC patients rate their quality of life (QoL) and cure rates differently than their younger counterparts, detailed information about long-term QoL after curative treatment is needed for this cohort to enable shared decision-making based on comprehensive prognostic and QoL data for aggressive cancer treatments of HNSCC patients [8,9,10,11]. Elderly HNSCCs patients weight treatment-related toxicities and use coping strategies in a different manner compared to younger patients [12], complicating the translation of QoL studies that included all patients irrespectively of their age. In addition, physicians often inapplicably extrapolate from treatment-related toxicities on patients’ general QoL, wherefore patient-reported QoL information are required [13,14].

The majority of studies which examined QoL of surviving elderly HNSCC patients focused on patients who underwent surgery, and the proportion of patients undergoing definitive (chemo)radiotherapy was relatively small [15,16,17,18,19]. There are only few studies that had a special focus on elderly HNSCC patients receiving (chemo)radiotherapy, and some of these studies incorporated patients with out-of-date radiotherapy techniques such as 2D or 3D radiotherapy treatments [20,21]. Furthermore, many studies focused on peritherapeutic QoL alterations, and the information about the long-term effects of radiotherapy treatment on patients’ QoL in the elderly population are limited [22].

We therefore aimed to examine the long-term QoL of surviving elderly HNSCC patients after curative definitive or adjuvant (chemo)radiotherapy in a large tertiary cancer center.

## 2. Results

### 2.1. Long-Term QoL Is Comparable between Surviving Elderly HNSCC Patients and the General Population

**Figure 1:**
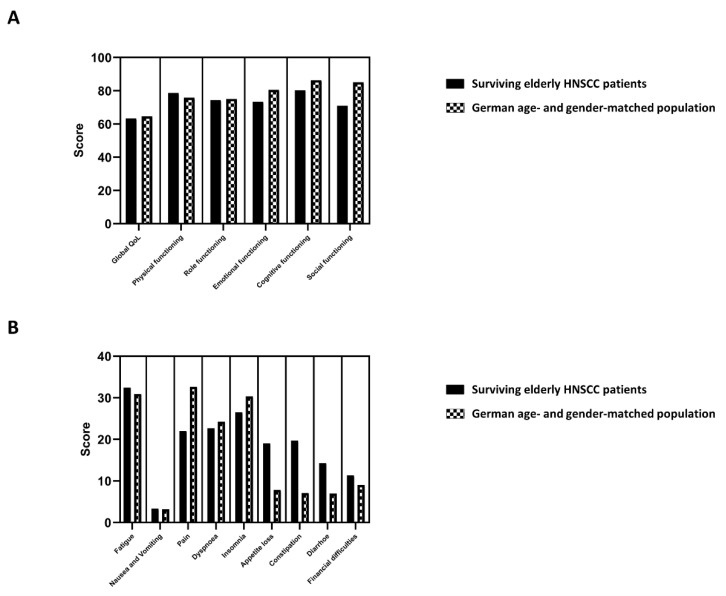
Comparison between surviving elderly HNSCC (head-and-neck squamous cell carcinoma) patients and matched patients from the German general population (with the same age and gender) regarding the functioning subscales (**A**) and the symptom subscales/items (**B**) of the EORTC QLQ-C30 questionnaire. Bars represent the mean values of the different subscales. General population norm data for Germany are obtained from [23].

The EORTC QLQ-C30 containing 5 functional scales (physical, role, cognitive, emotional and social), 3 symptom scales (fatigue, pain and nausea/vomiting), a global QoL scale and 6 single items (dyspnea, insomnia, appetite loss, constipation, diarrhea and financial difficulties) was used to assess patient-reported QoL in surviving elderly HNSCC patients after (chemo)radiotherapy. In order to compare the global QoL as well as the functional and symptom EORTC QLQ-C30 subscales of our elderly HNSCC cohort with the general German population of the same age and gender, we used the recently published data of the EORTC QoL Group incorporating the data of about 1000 German people [23]. The median/mean global QoL of our elderly HNSCC patient cohort amounted to 66.7/63.4 points, while the median/mean global QoL of the matched German population was 65.3/64.6 points (Figure 1A). The largest difference between our cohort consisting of elderly surviving HNSCC patients and the German reference population was observed regarding the domain of social functioning: Here, the mean value was 14.2 points higher in the age- and gender-adjusted German population (85.2 versus 71.0), which is considered a clinically meaningful difference and indicates an impaired social functioning of surviving elderly HNSCC patients after (chemo)radiotherapy. The remaining functional subscales did not show clinically meaningful differences between elderly HNSCC patients and the age-adjusted general population. Additionally, we compared the individual symptom subscales/items of the EORTC QLQ-C30 between both cohorts (Figure 1B): Here, appetite loss (19.0 versus 7.8) and constipation (19.7 v.s. 7.1) were more apparent in surviving elderly HNSCC patients compared to the general population. Interestingly, pain was in turn less pronounced in elderly HNSCC patients (22.0 v.s. 32.6).

### 2.2. HPV Positivity and Performance Status Correspond with Long-Term QoL in Surviving Elderly HNSCC Patients

**Figure 2:**
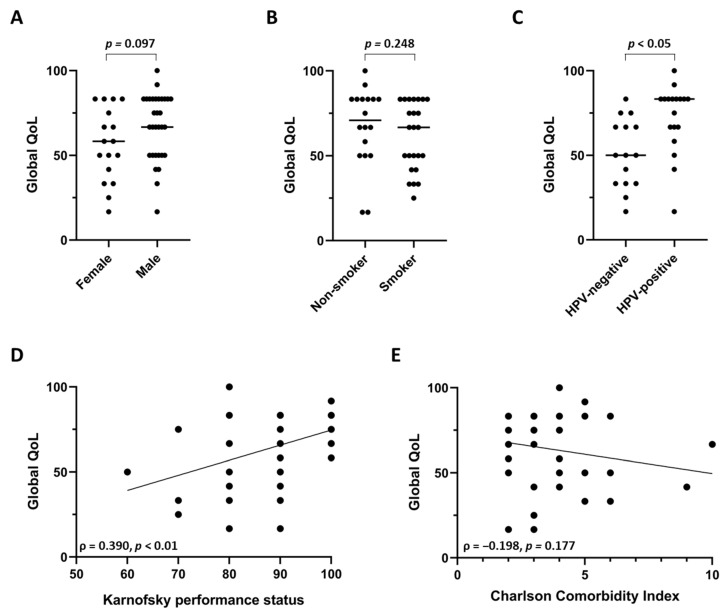
Global QoL of surviving elderly HNSCC patients undergoing (chemo)radiotherapy in terms of patients’ gender (**A**), smoking status (**B**) and HPV status (**C**). Correlation between global QoL and Karnofsky performance status (**D**) or Charlson Comorbidity Index (**E**). (**A**–**C**) Each scatter plot represents one patient, and the line shows the median value. Groups were compared using Mann-Whitney U tests. (**D**,**E**) Spearman’s rho with the corresponding *p*-value is indicated for the correlation analyses. The line shows the regression line of the xy-matrix.

We examined potential influences of patients’ gender, smoking status and HPV positivity on the long-term QoL of surviving elderly HNSCC patients. There was a trend towards decreased global QoL in female patients (*p* = 0.097, Mann-Whitney U test) (Figure 2A), which can also be observed within the general population. While the median global QoL was comparable between smokers and non-smokers (*p* = 0.248, Mann-Whitney U test) (Figure 2B), elderly patients with HPV-positive tumors exhibited considerably increased global QoL values status (*p* < 0.05, Mann-Whitney U test) (Figure 2C). The median/mean global QoL was 50.0/51.1 points in HPV-negative HNSCC patients, while it amounted to 83.3/72.2 points in HPV-positive patients. The baseline performance status prior to the initiation of radiotherapy was observed to moderately correlate with the global QoL of the surviving patients (ρ = 0.390, *p* < 0.01) (Figure 2D), whereas such an association could not be demonstrated for the patients’ comorbidity burden as measured by the age-adjusted Charlson Comorbidity Index (ρ = −0.198, *p* = 0.177) (Figure 2E).

### 2.3. Prior Tumor Resection and Concomitant Chemotherapy Do Not Influence Long-Term QoL in Surviving Elderly HNSCC Patients

**Figure 3:**
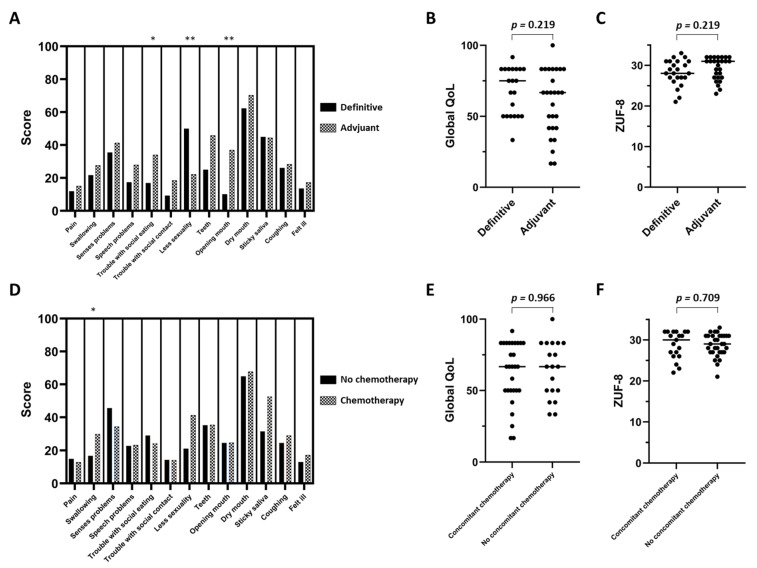
Comparison between the definitive and adjuvant (chemo)radiotherapy group regarding the different EORTC QLQ-H&N35 symptom subscales (**A**), general QoL (**B**) and patient satisfaction measured by the ZUF-8 questionnaire (**C**) of surviving elderly HNSCC patients. Influence of concomitant chemotherapy administration in surviving elderly HNSCC patients in terms of the EORTC QLQ-H&N35 symptom subscales (**D**), general QoL (**E**) and patient satisfaction (**F**). Groups were compared using Mann-Whitney U tests. * *p* < 0.05, ** *p* < 0.01.

Based on the EORTC QLQ-C30 and EORTC QLQ-H&N35 questionnaires, potential influences of tumor surgery prior to radiotherapy or the addition of concomitant chemotherapy on the long-term QoL were assessed. The majority of EORTC QLQ-H&N35 symptom subscales showed comparable score values between the definitive and adjuvant radiotherapy group; however, patients reported less symptoms regarding “troubles with social eating” (*p* < 0.05) and mouth opening (*p* < 0.01) without prior tumor resection (Figure 3A). In contrast, patients who received primary resection with adjuvant (chemo)radiotherapy had fewer problems with reduced sexuality than patients undergoing primary (chemo)radiotherapy (*p* < 0.01). Neither global long-term QoL nor patient satisfaction, as measured by the ZUF-8 questionnaire, differed significantly between both groups (*p* = 0.219 for both categories) (Figure 3B,C). The long-term swallowing function was the only reported symptom that significantly differed between the radio- and chemoradiotherapy group (*p* < 0.05, in favor of radiotherapy) (Figure 3D). In addition, concomitant chemotherapy administration had no long-term effect on the QoL (*p* = 0.966) and patient satisfaction (*p* = 0.709) of surviving elderly HNSCC patients (Figure 3E,F).

### 2.4. Long-Term QoL in Surviving Elderly HNSCC Patients Does Not Correlate with Physician-Assessed CTCAE-Based Chronic Toxicities

The vast majority of patients exhibited either no (*n* = 2, 4%) or only CTCAE (Common Terminology Criteria of Adverse Effects) grade 1/2 (*n* = 41, 82%) chronic toxicities (Table 1). A total of 7 patients (14%) suffered from severe (CTCAE grade 3) toxicities; however, there was no significant difference in terms of the long-term global QoL in dependence of the maximum physician-assessed chronic toxicity (*p* = 0.640, Kruskal-Wallis test). While the median global QoL amounted to 70.9 and 66.7 points in patients with grade 1 and grade 2 toxicities, respectively, it was 66.7 points in patients with grade 3 chronic toxicities. Similarly, patient satisfaction did not differ depending on patients’ chronic toxicities (*p* = 0.517, Kruskal-Wallis test).

The majority of surviving elderly HNSCC did not use analgesic medication (*n* = 40, 80%) or nutritional supplements (*n* = 40, 80%) within the last week prior to questionnaire completion, and this rate was comparable between the definitive and adjuvant (chemo)radiotherapy groups (Table 2). While none of the 23 patients in the definitive treatment cohort had a long-term feeding tube dependence, 4 of 27 patients in the adjuvant treatment group required a permanent feeding tube (*p* = 0.115, Fisher’s exact test).

## 3. Discussion

In this cross-sectional analysis including 50 surviving elderly HNSCC patients who received (chemo)radiotherapy at a large tertiary cancer center, we could demonstrate a global QoL that was comparable to the age- and gender-adjusted German general population. Furthermore, we did not observe statistically different global QoL values between definitive and adjuvant (chemo)radiotherapy or between radiotherapy and concomitant chemoradiotherapy. Surviving elderly HPV-positive patients were found to exhibit a superior QoL than their HPV-negative counterparts, which is an important fact regarding current de-escalation debates for HPV-positive patients. Overall, our findings may support clinicians in counselling elderly HNSCC patients for curative therapies regarding their long-term QoL.

Previous studies assessing patient-reported outcomes after HNSCC treatment could show equivalent health-related QoL compared to the age- and gender-matched population in younger patients, but to date, no information has been available for the distinct subgroup of elderly patients, for whom QoL is deemed considerably more important [24,25]. To the best of our knowledge, we present, for the first time, evidence that this observation of equivalent QoL values holds also true for the elderly HNSCC population treated by (chemo)radiotherapy. The subscale value for pain was even lower by more than 10 points in elderly HNSCC survivors than in the age- and gender-controlled general German population. While fatigue is a frequent long-term sequela for several cancer entities such as stomach, lung and breast cancer [26,27], it seems to be a less common patient-reported adverse event in surviving elderly HNSCC patients after (chemo)radiotherapy when comparing with the general population. In this context, it should be noted that most HNSCC patients adapt to their physical impairments such as xerostomia or dysphagia, leading to sometimes better global QoL values than physicians would assume. For instance, long-term QoL is not reduced after laryngectomy, contrary to common physician belief [28]. Although the global QoL as well as the physical, role, emotional and cognitive functioning were comparable between elderly HNSCC patients and the age- and gender-matched population, there was a considerable decrease of surviving HNSCC patients’ social functioning. Previous studies have also reported that social functioning was the worse functioning scale of surviving HNSCC patients after radiotherapy [20,21].

In a prospective study of Derks and coworkers, elderly HNSCC patients (≥70 years) exhibited comparable global QoL values to patients aged between 45 and 60 years at one year after treatment [17]. In contrast, Laraway and colleagues could demonstrate for surgical HNSCC treatments that several QoL domains such as physical and emotional function were superior in HNSCC patients aged 65 years and older compared to younger patients [19]. One hypothesis for these observations may be that elderly patients have less lifetime to lose and that their expectations regarding outcome are lower compared to their younger counterparts.

Interestingly, elderly HPV-positive HNSCC patients were found to have a considerably higher QoL than HPV-negative patients in our study. There are several possible explanations for this observation: In general, HPV-positive patients exhibit a higher socioeconomic status, increased financial income and higher education level which may all contribute to a usually superior QoL in this cohort [29]. In a substudy of the Trans-Tasman Radiation Oncology Group (TROG) 02.02 trial, HPV-positive oropharyngeal carcinoma patients were demonstrated to have elevated baseline QoL values but a more pronounced decline during chemoradiotherapy; however, they again had a better QoL than HPV-negative oropharyngeal cancer patients at 12 months after treatment [30]. This finding was confirmed in other analyses for long-term surviving patients with HPV-positive HNSCC [31,32]. The high global QoL results of surviving elderly HNSCC patients after (chemo)radiotherapy should be considered in the current treatment de-escalation debates.

In our analysis, simultaneous chemoradiotherapy resulted in similar global QoL, head-and-neck cancer-related symptom severity and patient satisfaction compared to patients receiving radiotherapy alone. Chemotherapy administered either in the definitive setting or postoperatively due to positive resection margins or extranodal extension remains controversial, especially in the postoperative setting. Neither the EORTC 22931 nor the RTOG 9501 trials that established the criteria for postoperative chemoradiotherapy did provide sufficient evidence for the usage of concomitant chemotherapy in the elderly, either due to complete exclusion of patients above 70 years (EORTC 22931) or due to low numbers (*n* = 25) of patients aged 70 years or older (RTOG 9501) [33,34]. The previous retrospective National Cancer Data Base (NCDB) and the Medicare-linked Surveillance, Epidemiology, and End Results (SEER) database analyses could not demonstrate a benefit for adding chemotherapy to postoperative radiotherapy in the case of positive resection margins or extranodal extension [35,36,37]. Our results revealed at least no signs of significantly deteriorated long-term QoL in surviving elderly HNSCC patients after chemoradiotherapy; only patient-reported swallowing dysfunction was found to be significantly higher (and clinically significant with a difference >10 points) in the chemoradiotherapy group. This finding is in line with a previous study in which patients undergoing chemoradiotherapy exhibited swallowing disorders more frequently than patients treated by radiotherapy alone [38].

In our analysis, we could not detect a significant difference regarding patients’ long-term global QoL in dependence of the treatment modality. However, social eating as well as mouth opening showed clinically meaningful and significantly preferable values for the definitive (chemo)radiotherapy group which is in line with findings of other studies that reported superior QoL, in particular swallowing, social eating, speech and taste of patients undergoing definitive (chemo)radiotherapy compared to patients treated by surgery plus adjuvant (chemo)radiotherapy [39,40]. It should be also emphasized that results of previous studies concerning the long-term QoL of HNSCC patients after radiotherapy are difficult to transfer to the current situation, in which high-precision intensity-modulated and image-guided radiotherapy define the state-of-the art treatment. For instance, Huang et al. could prove the superiority of these modern techniques in regards to a better swallowing-related QoL of surviving HNSCC patients [20].

No association between physician-assessed chronic toxicities according to CTCAE and global QoL in our cohort of surviving elderly HNSCC patients could be observed. There were patients with only mild or moderate chronic toxicities (CTCAE grade 1–2) and a considerably reduced long-term global QoL, and vice versa, patients with severe chronic toxicities (CTCAE grade 3) were found to exhibit very high QoL scores. The DAHANCA group compared physician-reported and patient-reported outcome measures and observed a significant correlation of some observer-based toxicities such as dysphagia with patient-reported global QoL, whereas other chronic toxicities such as fibrosis and oedema did not correlate with patients’ global health status [41]. It has been reported that physicians commonly focus on higher-grade (CTCAE grade ≥ 3) toxicities and underestimate the cumulative impact of lower-grade toxicities on the patient-reported health status [42]. Following the promising feasibility and clinical results from prospective trials that investigated patient-reported outcomes, many institutions are currently integrating patient-reported outcome measures into the clinical routine, and previous studies also demonstrated the feasibility to perform app-based electronic patient-reported outcome measures in HNSCC patients [43,44,45,46].

Despite providing important information for clinicians regarding the long-term QoL of surviving elderly HNSCC patients after radiotherapy, our analysis has some limitations. Due to the cross-sectional study design, we were not able to longitudinally assess patients’ QoL and compare to baseline values prior to radiotherapy. In addition, patients who agreed to participate in the study may exhibit differences in their QoL compared to patients who declined participation (*n* = 7) or failed to return the questionnaire (*n* = 4). We furthermore only focused our analysis on patients who were free from active cancer and did not receive any antineoplastic treatment, so the generalizability to patients with persistent or recurrent disease is limited. Mild-to-moderate toxicity rates are known to be underreported in retrospective assessments compared to prospective trials, which is a caveat of our study; in addition, the exact onset of these toxicities could not be accurately assessed due to the cross-sectional character of this analysis. At last, it should be acknowledged that extrapolation of our results to the situation in other countries is not obvious, as patient-reported QoL data are subject to considerable intercultural and interethnic differences [47,48], pointing out the necessity for multinational studies in the future.

## 4. Materials and Methods

### 4.1. Patients and Treatment

The Independent Ethics Committee of the University of Freiburg approved this cross-sectional study in advance (reference no. 371/20), and written informed consent was obtained from all patients who agreed to participate.

**Figure 4:**
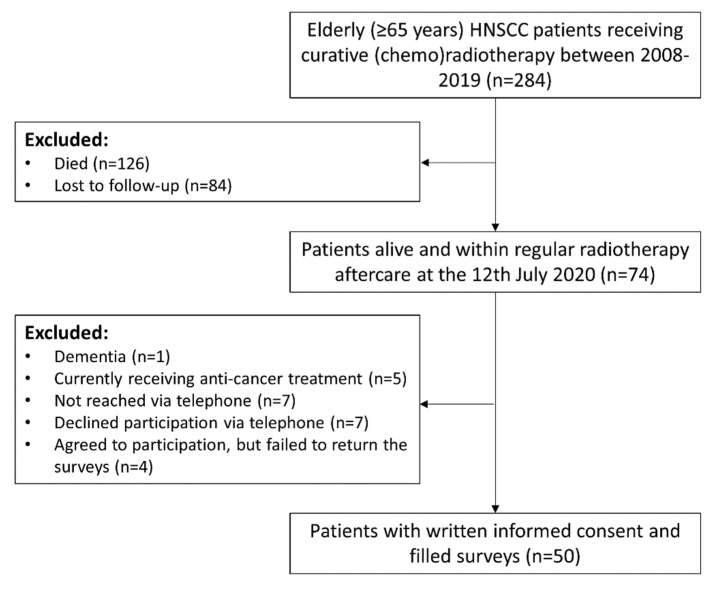
CONSORT flow diagram showing the enrollment of participants in the study.

HNSCC patients aged ≥65 years who were treated with curative (chemo)radiotherapy between 2010 and 2019 at the Department of Radiation Oncology, University of Freiburg Medical Center, and who were still alive at one year after radiotherapy and not lost to follow-up, were eligible for this study. Seventy-four patients met the inclusion criteria and we attempted to inform them about the QoL study. Fifty-four patients agreed to participate, of which 50 answered the required questionnaires. The CONSORT diagram for the patient screening is demonstrated in Figure 4.

**Figure 5:**
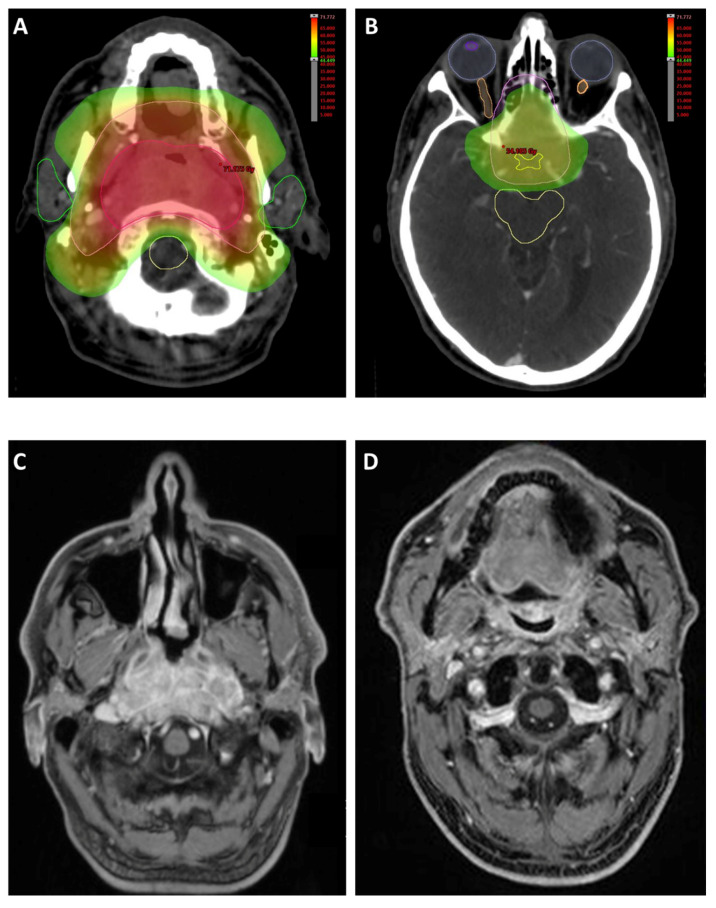
67-year-old male patient with an EBV-positive nasopharyngeal squamous cell carcinoma, infiltrating the sella turcica, clivus and right-sided internal carotid artery and affecting the chiasma, cT4 cN0 cM0, G3. Definitive cisplatin-based chemoradiotherapy was applied between May and July 2018. The primary tumor received 69.4 Gy in 37 fractions (14 fractions with 2 Gy and 23 fractions with 1.8 Gy). (**A**,**B**) Axial images showing bilateral parotid gland and spinal cord protection. (**C**) Contrast-enhanced MRI showing the nasopharyngeal carcinoma prior to radiotherapy. (**D**) Follow-up image at 2.5 years later (September 2020) showing no signs of recurrence.

Demographic and treatment characteristics of these patients were obtained from the electronic patient records. Patients with a smoking history of at least 10 pack/years were considered as smokers. Treatment decisions for all HNSCC patients were based on multidisciplinary tumor board recommendations. Patients who received definitive (chemo)radiotherapy received doses ranging at 70 Gy_EQD2_ to the primary tumor, whereas patients who received adjuvant (chemo)radiotherapy received 60–66 Gy_EQD2_ to the tumor bed. Photon radiotherapy was carried out using intensity-modulated radiotherapy, volumetric modulated arc therapy or tomotherapy (Figure 5).

In case of no medical contraindications, definitive radiotherapy was accompanied by concurrent chemotherapy for all patients with locally advanced HNSCC in the definitive treatment situation. Patients who were treated with primary surgery received concomitant chemoradiotherapy in the event of incomplete resection or extranodal extension.

### 4.2. Toxicity Analyses

Chronic treatment-related toxicities were classified in accordance with the Common Terminology Criteria of Adverse Effects (CTCAE) version 5.0. All toxicities occurring later than 90 days after radiotherapy were considered as chronic toxicities. Feeding tube dependence, usage of pain killers and intake of nutrition supplements were extracted from the EORTC H&N35 module.

### 4.3. Patient and Tumor Characteristics

A total of 50 patients agreed to participate in this study and filled out paper-based QoL and patient satisfaction questionnaires. The median age of the cohort at the time of treatment initiation was 69 years (range 65–82 years), and the majority of patients were male (*n* = 32, 64%). Most patients had a Karnofsky Performance Status between 90% and 100% (*n* = 33, 66%) at the time of radiotherapy, and the comorbidity burden, quantified by the age-adjusted CCI, was modest and shown by a median CCI value of 3 (range 2–10). Tumors were most commonly located in the oropharynx (*n* = 26, 52%), followed by the oral cavity (*n* = 9, 18%) and the larynx (*n* = 6, 12%). Twenty-three patients (46%) underwent definitive (chemo)radiotherapy (20 with definitive chemoradiotherapy and 3 with definitive radiotherapy), while 27 patients (54%) received (chemo)radiotherapy after primary surgery. Of these 27 patients, 16 received adjuvant radiotherapy, while 11 underwent adjuvant/additive chemoradiotherapy either due to R1 resection (*n* = 1) or due to extranodal extension (*n* = 11; one patient exhibited both R1 resection and extranodal extension). As described previously, completion rates for both radiotherapy and chemotherapy were very high with 96% and 97%, respectively [49,50]. Detailed patient and treatment characteristics can be found in Table 3.

### 4.4. QoL and Patient Satisfaction Questionnaires

Long-term QoL was assessed with the EORTC QLQ-C30 questionnaire (version 3) and the EORTC H&N35 module, while patient satisfaction was quantified using the ZUF-8 survey. The EORTC questionnaires were analyzed according to the EORTC scoring manual, while the ZUF-8 questionnaire as instrument for patient satisfaction with the treatment was examined as described by Schmidt et al. [51]. The ZUF-8 is the German adaption of the American CSQ-Questionnaire and consists of 8 questions with a 4-point Likert scale each, so that a score between 8 (minimal patient satisfaction) and 32 points (maximum patient satisfaction) can be achieved.

### 4.5. Statistical Analysis

Fisher’s exact tests were carried out to test for potential differences within contingency tables, and either Mann-Whitney U tests (2 groups) or Kruskal-Wallis tests (≥3 groups) were performed to compare QoL outcomes. Correlative analyses were conducted using Spearman’s rank correlations. Statistical significance was assumed for *p <* 0.05 throughout the study. IBM SPSS Statistics software version 25 (IBM, Armonk, NY, USA) was used for statistical analyses, and results were visualized using GraphPad Prism software version 8 (GraphPad Software, San Diego, CA, USA).

## 5. Conclusions

In this cross-sectional study involving 50 surviving elderly HNSCC patients who underwent radiotherapy at a large tertiary cancer center, we could demonstrate that patients’ self-reported long-term QoL is comparable to the age- and gender-adjusted general population. Neither the choice of primary curative treatment nor addition of concomitant chemotherapy did significantly deteriorate patients’ long-term global QoL or patient satisfaction. Interestingly, we could identify initial Karnofsky performance status and HPV status as parameters that were associated with post-treatment global QoL. The global QoL of surviving elderly HPV-positive HNSCC patients after radiotherapy was excellent (median global QoL = 83.3), which should be considered when discussing age-dependent treatment de-escalation for elderly HPV-positive HNSCC patients.

## Figures and Tables

**Table 1:** Median global QoL and patient satisfaction in dependence of the highest-graded chronic toxicity according to CTCAE (Common Terminology Criteria of Adverse Effects) version 5.0. *p*-values are derived from Kruskal-Wallis tests.

	Global QoL (QLQ-C30)	*p*-Value	ZUF-8	*p*-Value
0 (*n* = 2)	66.7		26.5	
1 (*n* = 14)	70.9		30.5	
2 (*n* = 27)	66.7		29.0	
3 (*n* = 7)	66.7	0.640	29.0	0.517

**Table 2:** Comparison between the definitive and adjuvant (chemo)radiotherapy group in terms of pain killer usage, nutritional supplement intake and feeding tube dependence (within the last week) among surviving elderly HNSCC patients. *p*-values are obtained from Fisher’s exact tests.

	Definitive (chemo)radiotherapy	Adjuvant (chemo)radiotherapy	*p*-Value
No pain killer usage	19	21	
Pain killer usage	4	6	0.736
No nutritional supplement intake	19	21	
Nutritional supplement intake	4	6	0.736
No feeding tube dependence	23	23	
Feeding tube dependence	0	4	0.115

**Table 3:** Patient and treatment characteristics of the study cohort consisting of 50 elderly HNSCC patients treated by (chemo)radiotherapy in our institution between 2010 and 2019. CCI = Charlson Comorbidity Index, HPV = Human papillomavirus.

Parameter		Median (Range)	
Age at Radiotherapy		69 Years (65–82)	
		*n*	%
Gender	male	32	64
	female	18	36
Smoking	non-smoker	19	38
	smoker	24	48
	missing	7	14
Karnofsky performance status	100%	8	16
	90%	25	50
	80%	13	26
	70%	3	6
	60%	1	2
CCI	2	10	20
	3	17	34
	4	9	18
	5	5	10
	6	7	14
	9	1	2
	10	1	2
Localization	nasopharynx	1	2
	oropharynx	26	52
	hypopharynx	1	2
	oral cavity	9	18
	larynx	6	12
	multi-level	3	6
	others	4	8
T stage	T1	12	24
	T2	17	34
	T3	12	24
	T4	9	18
n stage	N0	16	32
	N1	11	22
	N2	18	36
	N3	5	10
Grading	G1	2	4
	G2	23	46
	G3	24	48
	missing	1	2
HPV	HPV-negative	16	32
	HPV-positive	18	36
	missing	16	32
Radiotherapy completion	completed	48	96
	discontinued	2	4
Chemotherapy completion (*n* = 31)	completed	30	97
	discontinued	1	3
Treatment concept	definitive radiotherapy	3	6
	definitive chemoradiotherapy	20	40
	adjuvant radiotherapy	16	32
	adjuvant chemoradiotherapy	11	22
Chemotherapy (*n* = 31)	platinum agents	29	94
	nivolumab	2	6
		**Median (range)**	
Radiation dose (definitive treatment) in EQD2		70 Gy (55–72)	
Radiation dose (adjuvant treatment) in EQD2		66 Gy (59–66)	

## Data Availability

The anonymized data presented in this study are available on request from the corresponding author. The data are not publicly available due to data protection principles.
